# Suicide Risks Among U.S. College Students: a Time-Series Cross-Sectional Study Examining Institutional Characteristics and Behavioral Factors

**DOI:** 10.1007/s11121-025-01854-3

**Published:** 2025-11-21

**Authors:** Li Deng, Chanam Lee, Sungmin Lee, Yizhen Ding, Galen Newman

**Affiliations:** Department of Landscape Architecture and Urban Planning, College of Architecture, Texas A&M University, College Station, TX 77843 USA

**Keywords:** College students, Suicidal behavior, Institutional characteristics, Physical activity, Social activity, Suicide prevention planning, COVID-19

## Abstract

Suicide has become the second leading cause of death among U.S. college students, exacerbated by COVID-19. A more comprehensive understanding of its risk factors can guide the development of effective prevention strategies tailored to this population. We employed a time-series cross-sectional approach and used the national survey data from the American College Health Association to examine the effects of institutional characteristics (school locale, region, size, and type) and behavioral factors (physical and social activities) on suicide risks among U.S. college students across different pandemic phases (before, early phase, and late phase). We also tested whether behavioral factors moderated the association between the pandemic period and suicide risk. After adjusting for confounders, multilevel regression results showed that (1) suicide risk increased during the pandemic, particularly in the late phase; (2) institutional characteristics, including geographic region and religious affiliation, as well as student behaviors such as physical and social activities, were significant predictors of suicide risks; and (3) behavioral factors moderated the pandemic’s effect on suicide risk, as shown by the role of social activities (e.g., team sports, socializing with friends) amplifying the effect, whereas spending time with family mitigated it. Because risk rose fastest where certain social activities intensified pandemic effects, campuses should prioritize structured, lower-risk social engagement, while pairing selective strategies in high-activity settings (e.g., targeted screening, gatekeeper training) with universal measures (e.g., campus-wide suicide prevention program). Overall, this study offers evidence-informed guidance to shape campus environments that mitigate suicide risk and support student well-being, considering institutional context, behavioral factors, and public health crises.

**Supplementary Information:**

The online version contains supplementary material available at 10.1007/s11121-025-01854-3.

## Introduction

### Significance of the Problem

Suicide is a significant public health issue worldwide. In 2019, over 1.3% of all recorded deaths were attributed to suicide (World Health Organization, 2021). While global age-standardized suicide rates decreased by 36% from 2000 to 2019, the United States (U.S.) had a significant increase of 17% (World Health Organization, 2021). Suicide is particularly prevalent among youth and has become the second leading cause of death among U.S. college students (Turner et al., 2013). This has led to numerous problems in higher education, including increased demand for mental health services, decreased academic performance, disrupted campus communities, and heightened concern for student well-being (Drum et al., 2009; Eisenberg et al., 2007; Wilcox et al., 2010). Suicide extends far beyond the individual, reverberating across the entire campus community. Epidemiologic evidence suggests that each suicide affects a large network of around 135 people, many of whom may require support, and that more clusters can emerge without timely and coordinated postvention (Cerel et al., 2019). Specifically on college campuses, about 2 in 3 students report knowing someone who has attempted or died by suicide. Those bereaved by suicide in university communities face about a 10% absolute risk of a suicide attempt (Pitman et al., 2016). In addition, suicide and nonfatal self-harm carried an economic cost of over $500 billion in the U.S. in 2020 (Peterson et al., 2024), suggesting the need for substantial institutional resources for counseling, outreach, and coordinated postvention (CDC Suicide Prevention, 2024). These realities underscore universities as critical settings for systems-level prevention and postvention, aligning with the current CDC’s prevention guidance and national strategies (U.S. Department of Health and Human Services (HHS), 2024).

The recent pandemic has intensified this alarming trend. According to the American Foundation for Suicide Prevention (2024), the suicide rate among adolescents and young adults aged 15 to 24 rose from 13.95 to 15.15 deaths per 100,000 individuals between 2019 and 2021. This increase is likely attributable to the pandemic-related lockdowns and work-from-home policies (Loades et al., 2020). Although most colleges and universities have resumed pre-pandemic operations, the COVID-19 pandemic has left profound and lasting impacts on college students’ mental health. The unprecedented disruptions in academic routines, social life, and access to support services during this period amplified psychological distress and suicide risk among emerging adults (Deng et al., 2024). Examining this unique context offers critical insights into developing more comprehensive approaches to improve student well-being under conditions of crisis, while mitigating broader consequences for campus communities, healthcare costs, and U.S. society. These insights are not only important for understanding past impacts but also for informing more resilient mental health strategies that can prepare campuses for future public health emergencies or other systemic disruptions.

### Literature Review

Suicidal behavior is a complex issue influenced by various factors (Martinez-Líbano & Yeomans Cabrera, 2021). The “Ecological Model” proposed by McLeroy et al. (1988) emphasizes the significance of multi-dimensional factors, including intrapersonal, interpersonal, institutional, community, and public policy aspects, in designing effective interventions to address public health issues (McLeroy et al., 1988). While extensive research has explored intrapersonal and interpersonal predictors of suicide (e.g., mental illness, substance use, or social support) (Pillay, 2021), fewer studies have examined the roles of broader institutional characteristics and behavioral factors. Institutional characteristics refer to structural attributes of colleges and universities, such as geographic region, physical location, size, type, and institutional policies or regulations, that can influence students’ health issues (Deng et al., 2024; Oswalt et al., 2015) and the capacity of institutions to implement health promotion programs (Macri, 2019). Behavioral factors, on the other hand, reflect modifiable lifestyle patterns, including levels of physical and social activities, which are shown to significantly impact individual mental health and well-being (Kemel et al., 2022; Ono et al., 2011).

#### Institutional Characteristics and Suicide Risk

Despite the growing acknowledgment of contextual influences on student well-being, only a limited number of studies have examined how institutional characteristics shape suicide risk. For example, Daood (2009) found that Christian-affiliated institutions were associated with lower risks of suicidal ideation. Similarly, Johnson and Smith (2022) reported that participation in religious activities and commitment reduced suicidality among rural college students. These findings suggest the influential role of institutional culture and affiliation in influencing suicide risks, underscoring the need for greater attention to structural factors in suicide prevention research within higher education.

#### Behavioral Factors and Suicide Risk

Behavioral factors, particularly physical activity (PA) and social activity (SA), have been identified as protective against suicidal ideation and behavior (Calati et al., 2019; O’Connor, 2015). Empirical studies demonstrate that regular physical activity reduces suicidal ideation (Vancampfort et al., 2018) and enhances mental health (Petruzzello & Box, 2020) and overall well-being (Buecker et al., 2021). Likewise, social integration, including positive social connections with family, friends, and social groups, as well as a sense of community, helps mitigate suicidal ideation and behavior (Fässberg et al., 2012; King & Merchant, 2008). These behavioral dimensions are not only modifiable but also sensitive to institutional and environmental contexts, making them critical components of effective suicide prevention strategies.

### Study Objectives and Questions

Higher education institutions now face growing challenges in addressing student mental health and suicide behavior. Existing literature highlights the urgent need for a thorough examination of the multi-level risk factors to better inform institutional strategies and decisions. Therefore, this study examined the role of COVID-19 (before, early phase, and late phase), institutional characteristics (i.e., school locale, region, size, and type), and behavior factors (i.e., PA and SA) in influencing suicidal behavior among U.S. college students, while controlling for individual demographic characteristics and health conditions. This study aims to enhance the understanding of how institutional characteristics and behavioral factors affect student suicidal behavior under conditions of crisis. In addition, this study seeks to guide evidence-based campus-wide planning and management strategies to mitigate suicidal behavior in higher education and facilitate recoveries from COVID-19 syndrome. The following research questions guide this study.

1. How did the suicide risk and behavioral factors among college students change from pre-COVID-19 to early and later phases of COVID-19?
2. After adjusting for individual characteristics, how did COVID-19, institutional characteristics, and behavioral factors affect suicide risks?
3. How did physical and social activities moderate the COVID-19-suicidal risk relationships?

## Materials and Methods

### Data Source and Sample

This study utilized the American College Health Association’s National College Health Assessment (ACHA-NCHA) survey data, which is conducted every academic semester to examine students’ health habits, behaviors, and perceptions. The survey’s reliability and validity have been verified by the ACHA-NCHA Advisory Committee (American College Health Association, 2013). Since Spring 2000, over 2.2 million students from 1000 member schools have participated, with an annual response rate of 13%–14% at each school (American College Health Association, 2024). Before data cleaning, 4.52% of values were missing in the raw dataset. We assessed missingness by comparing demographic and institutional characteristics between respondents with complete versus incomplete data. The distributions were generally similar, providing no strong evidence that missingness was associated with suicide-related outcomes. In addition, individual samples that did not respond to the variables of interest were excluded. Following data screening and outlier removal (> ± 3 SD), 3.50% of cases were removed, which falls within an acceptable range for large-scale survey research (Dong & Peng, 2013). The final sample consisted of 250,385 students from 373 colleges who completed the survey between Fall 2019 and Spring 2022.

### Study Variables and Measures

**Fig. 1:**
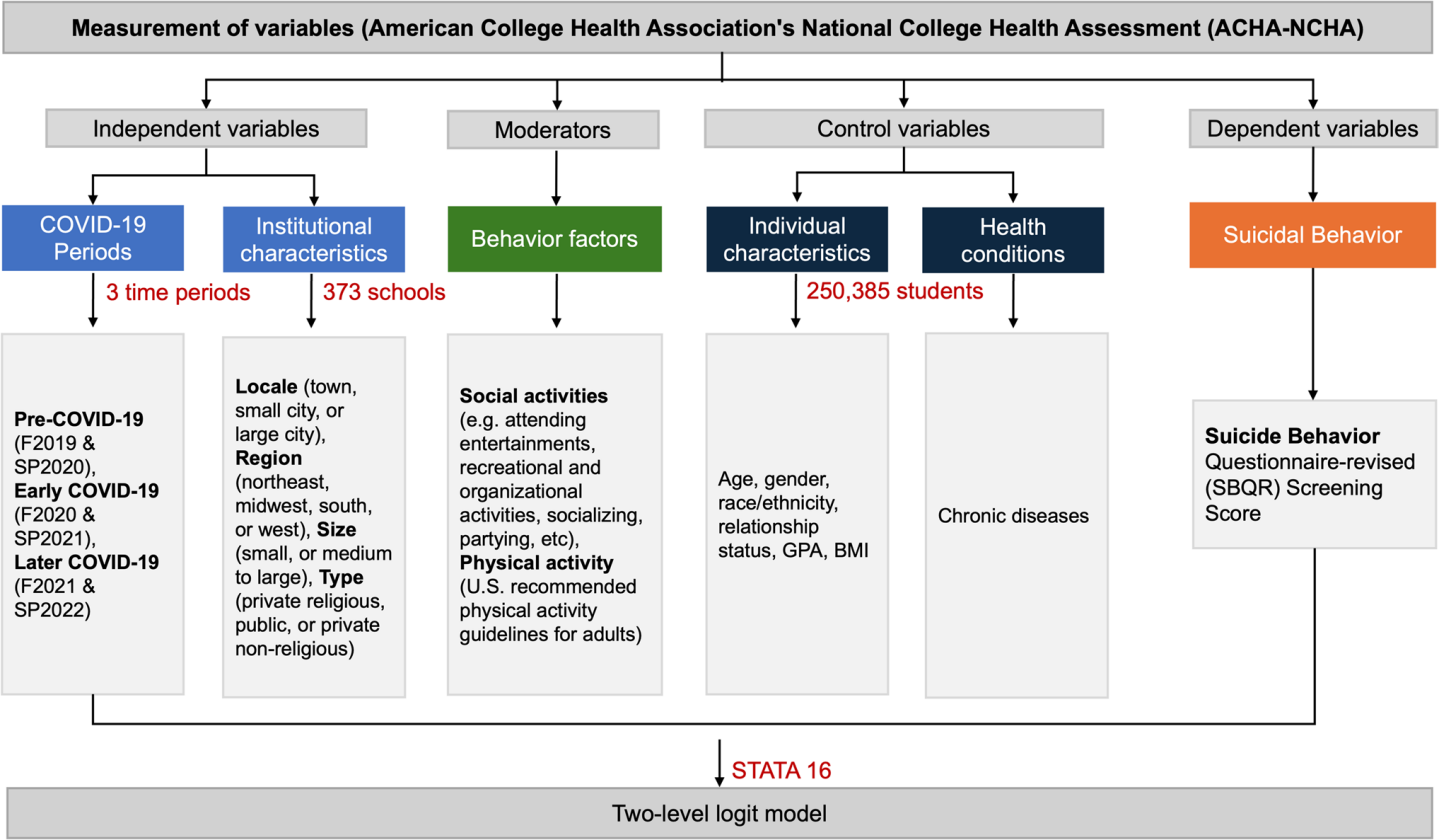
The research method framework of this study

Fig. 1 illustrates the research method framework of the study, and the details of the study variables are as follows.

#### Dependent Variable

##### Suicidal Behavior

College students’ suicide behavior was assessed utilizing the validated Suicide Behavior Questionnaire-revised (SBQR) scale (Osman et al., 2001), which comprises four items measuring different dimensions of suicidality. Following SBQR scoring criteria, the total score was calculated by summing the scores of each item, ranging from 3 to 18. For statistical analysis, the total score was recoded into a binary variable: scores of 7 or higher indicated a “positive screening—high suicide risk,” while scores of 6 or lower indicated a “negative screening—low suicide risk.”

##### Reliability of the SBQR Scale

This study calculated the reliability of the SBQR scale measuring suicidal behavior. The Cronbach’s alpha coefficients for the SBQR scale were 0.84, 0.84, and 0.85 before, early phase, and late phase, respectively*.* All Cronbach’s alpha values exceeded 0.80, indicating excellent internal consistency of the scale items across different stages of the pandemic (Landis & Koch, 1977).

#### Independent Variables

##### COVID-19

The ACHA-NCHA data was divided into different COVID-19 phases to evaluate its impact on suicide behavior: before (Fall 2019 and Spring 2020), early phase (Fall 2020 and Spring 2021), and late phase (Fall 2021 and Spring 2022) of the pandemic. We classified Spring 2020 as before COVID because the ACHA–NCHA is administered each semester, and the Spring 2020 wave included only responses collected before March 16, 2020, shortly after the U.S. national emergency declaration on March 13 and just before widespread campus shutdowns (Jee, 2020). Given this timing, the Spring 2020 dataset reflects little to no COVID-19 influence on student responses. The early pandemic phase was characterized by emergency countermeasures such as quarantine, physical separation, and city/school lockdown (National Center for Immunization, 2020). The late phase reflected the return to in-person learning and the lifting of most COVID-19 restrictions at U.S. universities (Parks, 2021).

##### Institutional Characteristics

Four institutional characteristics, including school locale, region, size, and type, were examined based on their availability in the ACHA-NCHA dataset and their relevance to student mental health and well-being (Deng et al., 2024). School locale refers to the institution’s location; categorized as “town” (urban cluster with populations of 2500–49,999), “small city” (urbanized areas with populations of 50,000–249,999), or “large city” (urbanized areas with populations over 250,000). School region denotes the geographic location of an institution within the U.S. (Northeast, Midwest, South, and West). School size is categorized as “small size” (enrollment of fewer than 5000 students) and “medium or large size” (enrollment of 5000 students or more), consistent with prior studies and the NCHA-III codebook. Lastly, the school type classifies the institution as public, private religious, or private non-religious.

#### Moderators

##### Physical Activity

This study assessed whether college students met the U.S.-recommended PA guidelines for adults utilizing the criteria of whether they engaged in at least 2 days of muscle-strengthening activity and had 150 or more minutes per week of moderate aerobic activity within the last 7 days (Olson et al., 2023). Participants’ responses were coded as a binary variable (no vs. yes).

##### Social Activity

The survey asked college students about their participation in the following activities during a typical week: (1) attending cultural events, movies, concerts, sports, or other entertainment with others; (2) participating in team sports, recreational sports, or physically active hobbies; (3) participating in student clubs or organizations; (4) socializing with friends; (5) partying; and (6) spending time with family. Each response was coded as a binary variable (no vs. yes). For statistical analysis, the SA score was calculated by summing the responses to each activity.

#### Control Variables

This study controlled for potential confounding variables identified in prior research and theoretical relevance to suicidal behavior among college students (Pillay, 2021). These included demographic and health-related characteristics such as gender, age, race/ethnicity, relationship status, grade point average (GPA), body mass index (BMI), and the presence of chronic diseases. Chronic health diseases include chronic mental health disorders (e.g., anxiety, depression, posttraumatic stress disorder), cardiovascular diseases (e.g., heart and vascular disorders, high blood pressure), behavioral risk factors (e.g., alcohol or other drug-related abuse or addiction), and other diseases (e.g., cancer, diabetes, HIV/AIDS).

### Statistical Analysis

After checking normality and multicollinearity, we conducted multiple statistical analyses in STATA16 following the steps: (1) chi-squared tests to examine the differences in the levels of suicide risk (low vs. high) and PA/SA participation (no vs. yes) across three different COVID-19 periods; (2) a multilevel logit model to examine the main effect of COVID-19, institutional characteristics (i.e., school locale, region, size, and type), and behavioral factors (i.e., PA and SA) on suicide risks; and (3) another multilevel logit model to further assess the moderating role of PA/SA in the relationship between COVID-19 and suicide risks. This study employed multilevel logit modeling to examine both individual-level and institutional-level effects on suicide behavior, as well as to estimate the random intercept effects of student suicide behaviors across different institutions.

## Results

### Sample Characteristics

**Fig. 2:**
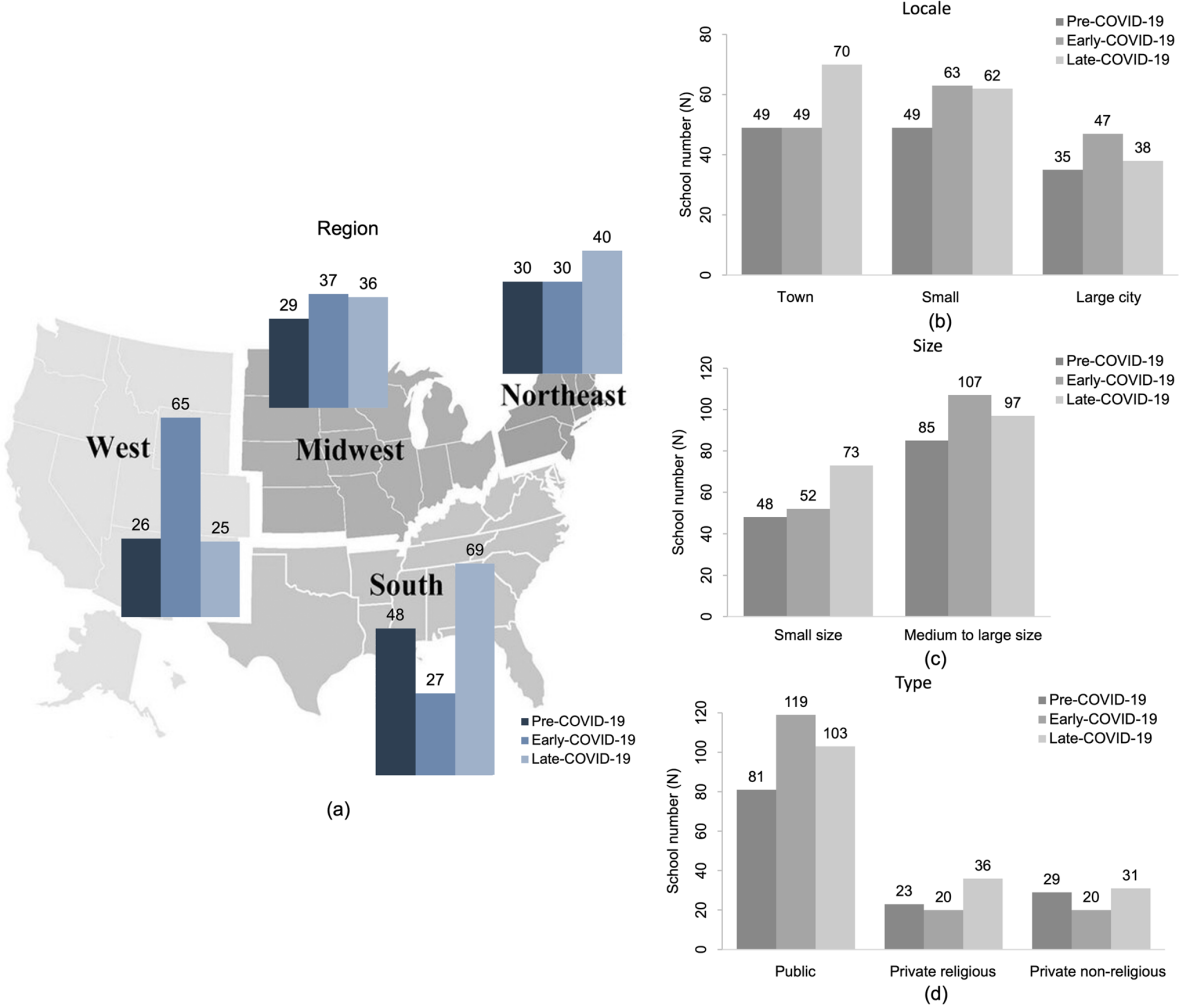
Characteristics of study colleges across COVID-19 periods. Panels display the distribution of colleges by (**a**) geographic region, (**b)** locale, (**c)** enrollment size, and (**d**) institutional type during the pre-, early-, and late-COVID-19 phases

The sample sizes of students (74,419, 92,883, and 83,083) and institutions (133, 159, and 170) varied somewhat across the three phases: pre-COVID-19, early-COVID-19, and late-COVID-19. Despite these differences, the repeated observations over time allow for both cross-sectional and longitudinal analyses, providing valuable insights into temporal trends and institutional influences. The study participants presented consistent distributions in socio-demographic factors and chronic health conditions across the different pandemic periods. Regarding study colleges, multiple schools participated in the survey study repeatedly over the three-year study period. As shown in Fig. 2a–d, most of the schools were from the southern region, were located in urban areas (i.e., small and large cities), had either a medium or large enrollment size, and were public institutions. Although institutional participation varied across regions during different phases of the pandemic, with more Western schools represented in the early phase and more Southern schools in the late phase, COVID-19 and region fixed effects were included in all multi-level regression models to account for these differences in estimating suicide risk. More details of the study characteristics of participants and colleges can be found in Appendices A and B.

### Changes in Suicidal Risks and Behavioral Factors Across Different COVID-19 Periods

Table 1 shows the changes in participants’ suicide risks as well as their physical and social activities across different COVID-19 periods. Students’ suicide risks varied significantly across three pandemic phases (*p* <.001), with a higher proportion of students reporting high suicide risks during the pandemic. Significant differences in all types of students’ physical and social activities were also observed across the three pandemic periods (*p* <.001). The decline was particularly significant in social activities such as attending movies, concerts, and other entertainment events (from 80.84% pre-COVID-19 to 53.91% during early COVID-19) and partying (from 44.31% pre-COVID-19 to 24.58% during early COVID-19). Conversely, there was a significant increase in students spending time with family, rising from 57.70% pre-COVID-19 to 74.16% during early COVID-19.

**Table 1:** Suicide risk and behavioral factors across different COVID-19 periods^*^

	Pre-COVID-19	Early-COVID-19	Late-COVID-19	*X*^*2*^	*P*-value
	*N* (%)	*N* (%)	*N* (%)		
**Suicidal behavior**					
Low suicide risk	56,036 (75.30)	68,603 (73.86)	60,459 (72.77)	130.54	<.001
High suicide risk	18,383 (24.70)	24,280 (26.14)	22,624 (27.23)		
**Behavioral variables**					
**Meet U.S. PA guideline**					
No	42,438 (57.03)	53,687 (57.80)	47,888 (57.64)	10.91	.004
Yes	31,981 (42.97)	39,196 (42.20)	35,195 (42.36)		
**Attend events with others**					
No	14,261 (19.16)	42,806 (46.09)	22,993 (27.67)	1.5e + 04	<.001
Yes	60,158 (80.84)	50,077 (53.91)	60,090 (72.33)		
**Participate in recreational sports**					
No	16,679 (42.94)	54,234 (58.39)	39,247 (47.24)	191.70	<.001
Yes	42,461 (57.06)	38,649 (41.61)	43,836 (52.76)		
**Participate in student clubs**					
No	31,958 (43.23)	63,097 (58.45)	46,877 (46.86)	4.3e + 03	<.001
Yes	49,438 (56.77)	44,845 (41.55)	53,159 (53.14)		
**Socialize with friends**					
No	3153 (4.24)	9352 (10.07)	4934 (5.94)	2.4e + 03	<.001
Yes	71,266 (95.76)	83,531 (89.93)	78,149 (94.06)		
**Party**					
No	41,445 (55.69)	70,049 (75.42)	48,891 (58.85)	8.4e + 03	<.001
Yes	32,974 (44.31)	22,834 (24.58)	34,192 (41.15)		
**Spend time with family**					
No	31,478 (42.30)	24,004 (25.84)	30,790 (37.06)	5.3e + 03	<.001
Yes	42,941 (57.70)	68,879 (74.16)	52,293 (62.94)		

^*^All are from the ACHA-NCHA data, and all are categorical variables; *PA* physical activity

### Multilevel Regression Model Results

Table 2 shows the multilevel logit model results. The low pseudo-*R*-squared score (0.18) is considered acceptable as most of the predictors in the model are statistically significant, and the objective of this study is to understand relationships between variables instead of estimating suicidal risks (Ozili, 2023). Although the intraclass correlation coefficient was small (.010), the school-level variance components were significant at the.05 level, indicating the necessity of employing multilevel modeling for data analysis (Bressoux, 2020).

**Table 2:** Multilevel regression results: effects of COVID-19, institutional characteristics, and behavioral factors on suicidal behavior

	Suicidal behavior		
	OR	*P* >|*z*|	95% CI
**COVID-19 variable**			
Pre-COVID-19 as reference			
Early-COVID-19	1.03^−^	.058	(0.99, 1.07)
Late-COVID-19	1.07^**^	<.001	(1.01, 1.31)
**Institutional variables**			
**Locale**			
Town (population < 49,999) as reference			
Small city (population 50,000–249,999)	1.01	0.72	(0.96, 1.06)
Large city (population > 250,000)	0.97	0.30	(0.92, 1.03)
**Region**			
Northeast as reference			
Midwest	1.14^**^	<.001	(1.06, 1.22)
South	1.06^−^	.083	(0.99, 1.13)
West	1.30^**^	<.001	(1.22, 1.40)
**Size**			
Small (< 5000 students) as reference			
Medium or large (5000 students or more)	0.98	0.41	(0.93, 1.03)
**Type**			
Private religious institution as reference			
Public institution	1.12^**^	.001	(1.05, 1.20)
Private non-religious institution	1.19^**^	<.001	(1.11, 1.29)
**Behavioral variables**			
Meet U.S. PA guideline^+^	0.84^**^	<.001	(0.82, 0.86)
Attend events with others^+^	0.83^**^	<.001	(0.81, 0.85)
Participate in recreational sports^+^	0.78^**^	<.001	(0.76, 0.80)
Participate in student clubs^+^	0.89^**^	<.001	(0.87, 0.91)
Socialize with friends^+^	0.78^**^	<.001	(0.75, 0.81)
Party^+^	0.93^**^	<.001	(0.91, 0.95)
Spend time with family^+^	0.73^**^	<.001	(0.71, 0.74)
**Individual variables**			
**Gender**			
Male as reference			
Female	0.97^**^	.002	(0.94, 0.99)
Non-binary	2.83^**^	<.001	(2.70, 2.96)
**Age**	0.96^**^	<.001	(0.95, 0.96)
**Race/ethnicity**			
Non-Hispanic white as reference			
Hispanic	0.98	.199	(0.95, 1.01)
Other minorities	1.21^**^	<.001	(1.18, 1.24)
**Relationship status**			
Not in a relationship as reference			
In a relationship	0.94^**^	<.001	(0.92, 0.96)
Married/partnered	0.81^**^	<.001	(0.77, 0.84)
**GPA**			
A as reference			
B	1.20^**^	<.001	(1.17, 1.23)
C and below	1.65^**^	<.001	(1.58, 1.71)
**BMI**			
Healthy weight as reference			
Underweight	1.12^**^	<.001	(1.08, 1.17)
Overweight	1.04^**^	.002	(1.02, 1.07)
Obesity	1.20^**^	<.001	(1.16, 1.23)
**Chronic disease**			
No chronic condition as reference			
Has one chronic condition	1.25^**^	<.001	(1.22, 1.29)
Has two or three chronic conditions	2.86^**^	<.001	(2.79, 2.93)
Has four or more chronic conditions	6.63^**^	<.001	(6.43, 6.84)
Pseudo-***R***-squared	0.18		
AIC	252,867.60		

^**+ **^No as reference group; ^**^a statistical significance at the 0.01 level (2-tailed); ^*^a statistical significance at the 0.05 level (2-tailed); ^−^a statistical significance at the 0.1 level (2-tailed); *PA* physical activity

#### Roles of COVID-19

COVID-19 had a significant impact on suicidal risks, consistent with the chi-squared results. Students faced significantly higher risks of suicide during the late pandemic phase (OR = 1.07,* p* <.001) and marginally higher risks during the early pandemic (OR = 1.03,* p* =.058), compared to the pre-pandemic period.

#### Roles of Institutional Characteristics

Institutional characteristics, including school region and religious affiliation, were significant predictors of suicidal risks. Students enrolled in institutions located in the Midwestern (OR = 1.14,* p* <.001), Southern (OR = 1.06,* p* < 0.10), and Western (OR = 1.30,* p* <.001) regions of the U.S. exhibited higher levels of suicidal risk compared to those in the Northeast region. Additionally, students attending public (OR = 1.12,* p* =.001) or private non-religious (OR = 1.19,* p* <.001) schools experienced increased suicidal risks compared to those in private religious institutions.

#### Roles of Behavioral Factors

Students’ behaviors (i.e., PA and SA) were significant predictors of suicidal risks. Students who met the recommended PA guidelines (OR = 0.84,* p* <.001) had lower suicidal risks compared to those who did not. Similarly, social activities, such as attending movies, concerts, or other entertainment with others (OR = 0.83), participating in team sports, recreational sports, or physically active hobbies (OR = 0.78), engaging in student clubs or organizations (OR = 0.89), socializing with friends (OR = 0.78), partying (OR = 0.93), and spending time with family (OR = 0.73) were negative predictors of suicide risks, all highly significant at the.001 level.

#### Moderating Effects of Behavioral Factors

**Fig. 3:**
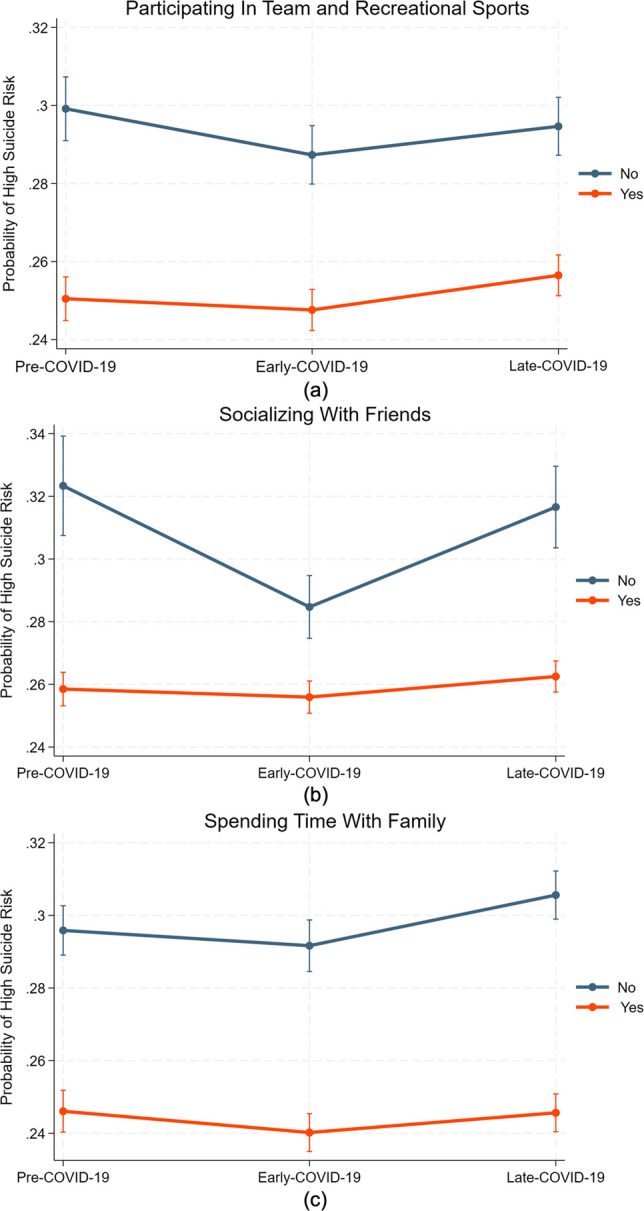
Moderation effect of social activities in COVID-19-suicide risk relationships. (**a)** probability of high suicide risk associated with participating in team and recreational sports; (**b)** probability of high suicide risk associated with socializing with friends; and (**c**) probability of high suicide risk associated with spending time with family

As shown in Table 3, the model results showed significant moderation effects of certain social activities. Students involved in team or recreational sports (OR = 1.06, *p* =.046) faced significantly higher suicidal risks during the late pandemic compared to those who did not (Fig. 3a). Students who reported socializing with friends at least once a week (OR = 1.22, *p* <.001) had higher suicidal risks during the early pandemic compared to those who did not (Fig. 3b). In contrast, students who were spending time with family (OR = 0.95,* p* =.036) experienced lower suicidal risks during the late pandemic compared to those who were not (Fig. 3c).

**Table 3:** Multilevel regression results: interactions of behavioral factors with COVID-19 on suicidal behavior

	Suicidal Behavior		
	OR	P>|z|	95% CI
**COVID-19 x meet U.S. PA guideline**			
Pre-COVID-19 × yes as reference			
Early-COVID-19 × yes	1.02	0.45	(0.97, 1.08)
Late-COVID-19 × yes	1.01	0.62	(0.96, 1.07)
**COVID-19 × attend events with others**			
Pre-COVID-19 × yes as reference			
Early-COVID-19 × yes	1.03	0.35	(0.97, 1.09)
Late-COVID-19 × yes	1.00	0.93	(0.94, 1.07)
**COVID-19 × participate in recreational sports**			
Pre-COVID-19 × yes as reference			
Early-COVID-19 × yes	1.05	0.10	(0.99, 1.12)
Late-COVID-19 × yes	1.06^*^	0.046	(1.00, 1.13)
**COVID-19 × participate in student clubs**			
Pre-COVID-19 × yes as reference			
Early-COVID-19 × yes	1.00	0.90	(0.95, 1.05)
Late-COVID-19 × yes	1.04	0.12	(0.99, 1.10)
**COVID-19 × socialize with friends**			
Pre-COVID-19 × yes as reference			
Early-COVID-19 × yes	1.22^**^	<.001	(1.10, 1.35)
Late-COVID-19 × yes	1.06	0.27	(0.95, 1.19)
**COVID-19 × party**			
Pre-COVID-19 × yes as reference			
Early-COVID-19 × yes	0.99	0.62	(0.93, 1.04)
Late-COVID-19 × yes	1.03	0.26	(0.98, 1.09)
**COVID-19 × spend time with family**			
Pre-COVID-19 × yes as reference			
Early-COVID-19 × yes	0.99	0.67	(0.94, 1.04)
Late-COVID-19 × yes	0.95^*^	0.036	(0.90, 1.00)
Pseudo-***R***-squared	0.19		
AIC	252,849.10		

^**^A statistical significance at the 0.01 level (2-tailed); ^*^a statistical significance at the 0.05 level (2-tailed); *PA* physical activity

## Discussion

Although most colleges and universities have resumed pre-pandemic operations, the COVID-19 period continues to serve as a critical lens for understanding persistent vulnerabilities in student mental health. By analyzing data collected during this distinct period, our study demonstrates that suicide risk among college students increased notably, particularly during the later stages of the pandemic. Concurrently, students reported significant declines in physical activity and various forms of social engagement, except spending time with family, suggesting shifts in behavioral patterns that may have compounded psychological distress (Elmer et al., 2020). These findings contribute to a growing body of evidence that contextualizes mental health within broader socioecological disruptions. Importantly, the observed trends underscore the need for adaptable and resilient suicide prevention strategies that extend beyond immediate crisis response. Rather than viewing the pandemic as an isolated event, it should be recognized as a stress test that revealed structural and behavioral vulnerabilities within higher education institutions (Browning et al., 2021). As such, colleges and universities must prioritize the development of proactive frameworks that address student mental health holistically, not only during public health emergencies but also in preparation for future systemic shocks, whether environmental, political, or social in nature. By embedding resilience into institutional planning and mental health programming, campuses can better safeguard the well-being of students in times of uncertainty and beyond (American Council on Education, 2020).

Moreover, this study demonstrates that institutional characteristics, including school region and religious affiliation type, impact suicidal behavior among college students. Students attending institutions located in the Midwestern, Southern, and Western U.S. are at higher suicide risk compared to their counterparts in the Northeastern region. This disparity may be due to socioeconomic and climatic differences across regions. Research has shown a relationship between regional suicide rates and socioeconomic factors, with regions of higher socioeconomic status generally experiencing lower suicide rates (Rehkopf & Buka, 2006). This aligns with the fact that the Northeastern region, which has the highest median household income in the country (Posey, 2023), tends to have lower suicide risks. Climatic factors may also contribute, as higher mean temperatures are associated with an increased incidence of suicide attempts among young people (Akkaya-Kalayci et al., 2017). Additionally, students attending non-religious public or private institutions demonstrated higher suicide risks than those at religiously affiliated institutions, supporting prior findings that religious affiliation may serve as a protective factor against suicidal ideation and behavior (Daood, 2009; Johnson & Smith, 2022). One explanation could be the enhanced sense of community and belonging as well as a shared value system fostered by rich cultural and religious activities in these institutions (Pargament et al., 2005; Smith & Snell, 2009), which may enhance coping mechanisms against suicidal tendencies.

Meanwhile, this study highlights the significant protective role of physical and social activities against suicidal behavior. College students who engage more frequently in physical and social activities, such as cultural events, sports, organizational activities, partying, and socializing with friends or family, exhibit lower suicidal risks compared to their less active peers. These findings are consistent with previous research identifying physical and social engagements as effective protective factors against suicide (Szanto & Whitman, 2021; Vancampfort et al., 2018). The protective effects of these activities are likely due to their many direct health-related benefits (Klussman et al., 2021; Monteiro et al., 2024), including reducing the risk of chronic diseases (Anderson & Durstine, 2019), improving mental health by alleviating anxiety and depression (McDowell et al., 2017), enhancing social interactions (Andersen et al., 2019), and strengthening a sense of community and belonging (Herbert, 2022; Thoits, 2011).

In addition, this study also explores the significance of physical and social activities in moderating the relationship between COVID-19 and suicidal behaviors. While prior literature has reported social engagement and team-based activities as protective against suicide risk (O’Connor, 2015), our results suggest that these factors may have had a different impact during the pandemic. This discrepancy may be attributed to the context of COVID-19, a highly contagious and globally disruptive pandemic (World Health Organization, 2020). Increased social contact and mobility during this period may have elevated infection and mortality risks, thus reversing the typically protective nature of social activities. It may also reflect the disruption, stress, and altered social dynamics associated with maintaining these activities under public health restrictions. Student-athletes and socially active students may have faced canceled events, limited group interactions, and disrupted routines, all of which can contribute to distress, especially when compounded by health-related anxiety or a perceived loss of identity, structure, and control (Collins et al., 2022; Graupensperger et al., 2020). These findings underscore the importance of contextualizing behavioral risk factors within broader social and institutional environments, particularly during times of crisis. Moreover, spending time with family during the pandemic is shown to reduce suicidal risks, aligning with previous research that underscores the protective role of social ties (Denney et al., 2009), emotional support (Wagner et al., 2003), and family cohesion (Prabhu et al., 2010) in mitigating suicide risks. These interesting findings highlight the differential roles that various kinds of SAs play in mitigating/aggravating suicidal behaviors during the pandemic.

A significant proportion of college students is at risk for suicidal behavior at the pre-pandemic baseline, with a significant escalation during the pandemic. Our findings highlight the complex, multifaceted influence of institutional characteristics and physical and social activities on suicidal behavior during health crises like the pandemic. These insights are crucial for the development of effective suicide prevention strategies and for managing public health emergencies, highlighting the need for tailored approaches that account for the timing and nature of interventions.

## Implications for Policies and Practice

This study advances the literature on suicide prevention in higher education by offering evidence-based insights into how both institutional characteristics and student behaviors influence suicide risk, particularly during large-scale disruptions such as the COVID-19 pandemic. By taking a multi-level perspective, the findings underscore the importance of aligning campus-level strategies with the broader social, behavioral, and structural contexts in which students are embedded. This suggests that one-size-fits-all approaches may fall short in addressing the diverse needs of student populations. Instead, suicide prevention efforts should be context-responsive and grounded in the unique characteristics of each campus environment.

Policymakers and campus health professionals should prioritize tailored interventions that reflect both institutional context and student group-specific risks. For example, institutions located in the Midwest, West, and South, or those classified as public or private non-religious, may require different resource allocation strategies compared to schools in the Northeast and religiously affiliated counterparts, given observed variations in suicide risk across institutional regions and types. Similarly, targeted outreach for student-athletes, students with disabilities, or those with limited social support may help address disproportionately elevated risk.

In parallel, since suicide risk increased in contexts where social activity patterns (e.g., team sports, socializing with friends) intensified the effects of the pandemic, campuses should prioritize structured, lower-risk forms of social engagement. There is a crucial need to design supportive campus environments that promote inclusive and safe social engagement, especially those that can be sustained during public health disruptions. To be effective, these efforts should combine selective strategies, such as targeted mental health screening and gatekeeper training in high social activity settings (e.g., athletic teams, residence halls, student organizations), with universal measures, including campus-wide suicide prevention programs, and 24/7 access to crisis support services.

Together, these findings offer actionable guidance for university stakeholders to integrate institutional contexts and behavioral insights into programmatic design, resource allocation, and policy development, ultimately contributing to healthier, more responsive campus environments that reduce suicide risk and support student well-being.

## Limitations and Future Studies

We acknowledge several limitations related to the data used in this study and propose directions for future research. First, the survey data are cross-sectional and collected at multiple time points, limiting the ability to draw causal inferences between institutional characteristics, behavioral factors, and suicidal risks. Future research should explore these relationships longitudinally using panel datasets. Second, response patterns in this study varied geographically over time, with greater representation from the Western U.S. during early COVID-19 and from the South during the later phase. Although COVID-19 and region fixed effects were included to mitigate potential confounding, we cannot fully exclude the influence of unmeasured regional or institutional factors that may have co-varied with pandemic timing and suicide risk. These considerations highlight the need for caution when interpreting the study’s findings. Third, the use of binary variables to represent physical and social activity may not adequately capture variations in frequency, intensity, or duration, potentially attenuating observed associations with suicide risk. Future research would benefit from incorporating more detailed or multidimensional measures of these behaviors. Fourth, while this study analyzed several new variables capturing institutional characteristics and behavioral factors not previously addressed, other factors, such as campus environments and healthcare facilities, may also potentially impact students’ suicide behaviors. Future research should explore these factors, especially those that are readily modifiable, to develop more effective prevention strategies in higher education. Finally, as our sample was limited to U.S. college students, future studies should incorporate a more diverse, international sample from various cultural backgrounds to improve external validity.

## Conclusion

Previous research on suicidal behavior has primarily focused on intrapersonal factors, with limited attention to the broader institutional and behavioral contexts that shape student mental health. This study contributes to the literature by demonstrating how both institutional characteristics and student behaviors are associated with suicide risk among U.S. college students, particularly in the context of the COVID-19 crisis. Specifically, we show that large-scale health crises like the pandemic can heighten suicide risks and that patterns of physical and social engagement may moderate these effects. In addition, our findings underscore the importance of developing context-responsive suicide prevention strategies in higher education. Policymakers and campus health professionals should consider institutional characteristics, regional context, and student behavior patterns when designing interventions. Promising interventions may include programs to promote safe physical and social engagement and tailored support services for students in higher-risk institutional settings. By linking institutional context with behavioral patterns, this study provides actionable insights to support the design of campus environments that reduce suicide risk and promote student well-being, especially during public health crises.

## Supplementary Information

Below is the link to the electronic supplementary material.

ESM 1 Supplementary Material 1 (DOC 65.5 KB)

## Data Availability

The data that support the findings of this study is available from the American College Health Association (www.acha.org/NCHA). This data is accessible for members of the ACHA through the completion of a request form available on the website noted above.
