# Supplementary material for: Suicide Risks Among U.S. College Students: a Time-Series Cross-Sectional Study Examining Institutional Characteristics and Behavioral Factors
Source: Prev Sci. 2025 Nov 21;26(8):1169–82. doi: 10.1007/s11121-025-01854-3 (PMC12804210; doi:10.1007/s11121-025-01854-3)
Supplement: Supplementary file 1 — Supplementary Material 1 (DOC 65.5 KB) [file 11121_2025_1854_MOESM1_ESM.doc]

**Appendix A**

Descriptive Statistics of Study Participants Included in the Before COVID-19, Early COVID-19, and Late COVID-19 Analysis*

|  | Pre-COVID-19 | Early-COVID-19 | Late-COVID-19 |  |
| --- | --- | --- | --- | --- |
|  | N (%) | N (%) | N (%) | |
| **N of PARTICIPANTS** | 74,419 (29.72) | 92,883 (37.10) | 83,083 (33.18) |  |
| **Gender** | | | | |
| Male | 24,167 (32.47) | 27,062 (29.14) | 26,459 (31.85) |  |
| Female | 47,795 (64.22) | 62,109 (66.87) | 52,107 (62.72) |  |
| Non-Binary | 2,457 (3.30) | 3,712 (4.00) | 4,517 (5.44) |  |
| **Age** ^c^ | 22.15 (5.05) | 23.22 (5.67) | 22.48 (5.47) |  |
| **Race/ethnicity** | | |  |  |
| Non-Hispanic white | 47,650 (64.03) | 54,128 (58.28) | 53,919 (64.90) |  |
| Hispanic | 10,283 (13.82) | 16,245 (17.49) | 10,731 (12.92) |  |
| Other minorities | 16,486 (22.15) | 22,510 (24.23) | 18,433 (22.19) |  |
| **Relationship status** | | |  |  |
| Not in a relationship | 39,143 (52.60) | 46,695 (50.27) | 43,443 (52.29) |  |
| In a relationship | 29,161 (39.18) | 36,000 (38.76) | 32,148 (38.69) |  |
| Married/partnered | 6,115 (8.22) | 10,188 (10.97) | 7,492 (9.02) |  |
| **GPA** | | | | |
| A | 44,269 (59.49) | 58,981 (63.50) | 53,741 (64.68) |  |
| B | 25,258 (33.94) | 28,785 (30.99) | 24,529 (29.52) |  |
| C and below | 4,892 (6.57) | 5,117 (5.51) | 4,813 (55.79) |  |
| **BMI** | | | | |
| Healthy weight | 42,678 (57.35) | 51,916 (55.89) | 46,704 (56.21) |  |
| Underweight | 3,661 (4.92) | 4,898 (5.27) | 4,511 (5.43) |  |
| Overweight | 17,402 (23.38) | 21,301 (22.93) | 18,852 (22.69) |  |
| Obesity | 10,678 (14.35) | 14,768 (15.90) | 13,016 (15.67) |  |
| **Chronic disease** | | | | |
| No chronic condition | 8,354 (38.10) | 34,457 (37.10) | 29,028 (34.94) |  |
| Has one chronic condition | 19,511 (26.22) | 22,755 (24.50) | 20,116 (24.21) |  |
| Has two or three chronic conditions | 18,114 (24.34) | 23,208 (24.99) | 21,367 (25.72) |  |
| Has four or more chronic conditions | 8,440 (11.34) | 12,463 (13.42) | 12,572 (15.13) |  |

Note. * All from the ACHA-NCHA data;

^C^ is reported as Mean (SD), and all others are reported as N (%).

**Appendix B**

Descriptive Statistics of Study Colleges Included in the Before COVID-19, Early COVID-19, and Late COVID-19 Analysis*

|  | Pre-COVID-19 | Early-COVID-19 | Late-COVID-19 |
| --- | --- | --- | --- |
|  | N (%) | N (%) | N (%) |
| **N of SCHOOLS** | 133 (28.79) | 159 (34.42) | 170 (36.80) |
| **Locale** |  |  |  |
| Town (population <49,999) | 49 (36.84) | 49 (30.82) | 70 (41.18) |
| Small city (population 50,000-249,999) | 49 (36.84) | 63 (39.62) | 62 (36.47) |
| Large city (population > 250,000) | 35 (26.32) | 47 (29.56) | 38 (22.35) |
| **Size** |  |  |  |
| Small (< 5,000 students) | 48 (36.09) | 52 (32.70) | 73 (42.94) |
| Medium or large (5,000 students or more) | 85 (63.91) | 107 (67.30) | 97 (57.06) |
| **Type** |  |  |  |
| Public institution | 81 (60.90) | 119 (74.84) | 103 (60.59) |
| Private religious institution | 23 (17.29) | 20 (12.58) | 36 (21.18) |
| Private non-religious institution | 29 (21.80) | 20 (12.58) | 31 (18.24) |
| **Region** |  |  |  |
| Northeast | 30 (22.56) | 30 (18.87) | 40 (23.53) |
| Midwest | 29 (21.80) | 37 (23.27) | 36 (21.18) |
| South | 48 (36.09) | 27 (16.98) | 69 (40.59) |
| West | 26 (19.55) | 65 (40.88) | 25 (14.71) |

*Note.* * All from the ACHA-NCHA data.
